# Human Papillomavirus Detection by Whole-Genome Next-Generation Sequencing: Importance of Validation and Quality Assurance Procedures

**DOI:** 10.3390/v13071323

**Published:** 2021-07-08

**Authors:** Laila Sara Arroyo Mühr, Daniel Guerendiain, Kate Cuschieri, Karin Sundström

**Affiliations:** 1 International HPV Reference Center, Department of Laboratory Medicine, Karolinska Institutet, SE-141 86 Stockholm, Sweden; sara.arroyo.muhr@ki.se; 2 Scottish Human Papillomavirus Reference Laboratory (SHPVRL), Laboratory Medicine, Royal Infirmary of Edinburgh, Edinburgh EH16 4SA, UK; Kate.Cuschieri@nhslothian.scot.nhs.uk; 3 School of Medicine, University of St Andrews, St Andrews KY16 9TF, UK; dgr7@st-andrews.ac.uk; 4 Department of Laboratory Medicine, Karolinska Institutet, SE-141 86 Stockholm, Sweden

**Keywords:** human papillomavirus, HPV, next-generation sequencing, NGS, whole-genome sequencing, WGS, deep sequencing

## Abstract

Next-generation sequencing (NGS) yields powerful opportunities for studying human papillomavirus (HPV) genomics for applications in epidemiology, public health, and clinical diagnostics. HPV genotypes, variants, and point mutations can be investigated in clinical materials and described in previously unprecedented detail. However, both the NGS laboratory analysis and bioinformatical approach require numerous steps and checks to ensure robust interpretation of results. Here, we provide a step-by-step review of recommendations for validation and quality assurance procedures of each step in the typical NGS workflow, with a focus on whole-genome sequencing approaches. The use of directed pilots and protocols to ensure optimization of sequencing data yield, followed by curated bioinformatical procedures, is particularly emphasized. Finally, the storage and sharing of data sets are discussed. The development of international standards for quality assurance should be a goal for the HPV NGS community, similar to what has been developed for other areas of sequencing efforts including microbiology and molecular pathology. We thus propose that it is time for NGS to be included in the global efforts on quality assurance and improvement of HPV-based testing and diagnostics.

## 1. Introduction

Tests for the detection of human papillomavirus (HPV) infection in humans have evolved dramatically over the last decades. Initial low-throughput hybridization/blotting techniques prefaced broad-spectrum signal amplification assays, which were then replaced by rapid high-throughput target-amplification assays involving quantitative polymerase chain reaction (qPCR). The latter tests are capable of detecting individual HPV-genotypes and have become the mainstay of HPV-based screening and clinical testing [1]. Arguably, the next “age” of HPV testing should involve going beyond simply detecting the presence or absence of HPV but, rather, providing more detailed insight into the likely course and clinical consequences of HPV infection.

Next-generation sequencing (NGS) of human or microbial genetic material is being applied increasingly in laboratory contexts, to facilitate research, population-based epidemiology, and recently, personalized patient diagnostics. NGS can be used as a highly sensitive method for HPV detection due to its ability to detect types at low copy number (even within multiple infections), novel types, and/or known types that are distantly related to primers/probes which may escape detection using standard molecular approaches [2,3,4]. When employing whole-genome NGS, which covers the entire genome and not only exomes or targeted regions, we conveniently allow high-accuracy determination of sequences below the phylogenetic level of genotype, i.e., variants and subvariants of HPV [3,4,5,6,7,8]. Indeed, in recent years, various studies have utilized NGS approaches to generate detailed insights into potential disease-related mechanisms of HPV. NGS has shown that certain sublineages of HPV are associated with a higher risk of cancer [9,10,11], and its high sensitivity also allows the attributable fraction of cervical cancer associated with HPV to be determined with greater precision compared to traditional PCR techniques [12,13]. Further, NGS has identified certain single-nucleotide polymorphisms (SNPs) associated with a higher likelihood of viral persistence [14] and the key role of HPV *E7* gene conservation in cervical cancer development [15].

Although NGS has been used in HPV research for some years with different applications, as described above, NGS in clinical diagnosis is not yet extensively used. Lack of standardization and quality guidelines, as well as expense and requirement for ancillary laboratory infrastructure, may have slowed down the adoption of this technology in clinical laboratories. However, as is discussed later, NGS use could ultimately improve diagnosis and management of patients with HPV-driven lesions. This includes utilities ranging from a more sensitive detection of HPV to detection of true viral persistence [13], identification of risk according to HPV sublineage [9,10,11], and detection of circulating HPV DNA in patients who have received cancer treatment [16].

NGS is a technology based on massively parallel sequencing or “deep sequencing” of nucleic acid sequences. Nucleic acid sequences are fragmented with each fragment being amplified and sequenced multiple times, providing a depth of information which can deliver accurate data at the nucleotide level. NGS can sequence the entire genome of HPV or be limited to specific areas of interest [11,14].

There are several general approaches to sequencing, depending on the size (i.e., length) of nucleotide reads obtained and detection method employed. Illumina and IonTorrent instruments obtain reads that are approximately 250 base pair (bp) long, whereas Oxford Nanopore MinIon and PacBio obtain longer reads, potentially exceeding 10,000 bp in length [17].

**Figure 1:**
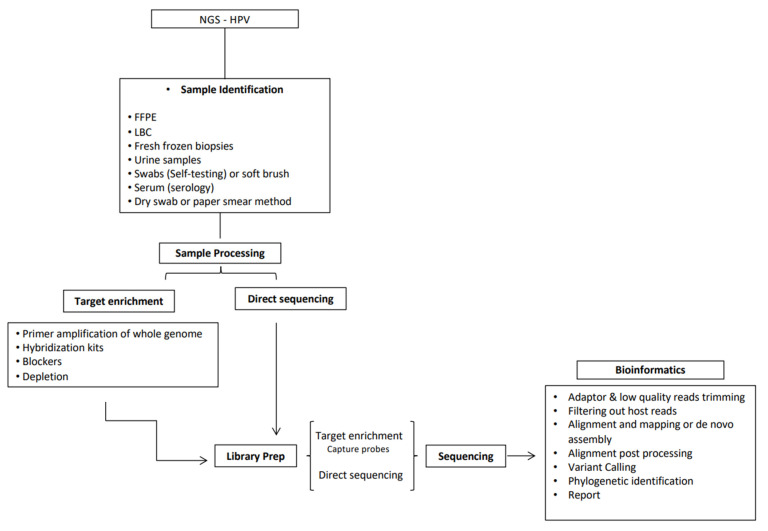
Next-generation sequencing (NGS) for human papillomavirus (HPV) detection and characterization process steps, from sample preparation to data analysis, with focus on whole-genome sequencing (WGS).

The whole NGS process requires several steps, which include initial sample identification, processing (nucleic acid extraction), viral enrichment (optional), library preparation, sequencing, and bioinformatic analysis of the raw data (Figure 1). While some of these steps are consistent with general requirements for molecular detection (i.e., sample extraction), the described downstream aspects arguably require an additional set of skills and analyses. Given the multistep nature of the process and the generation of large amounts of detailed data generated, it is essential that, where possible, standardization and quality checks to support consistency and integrity of data outputs are considered and implemented. For other applications, including those relevant to bacteriology and molecular pathology, quality guidelines have already been developed [18,19,20]. However, these are still lacking for the HPV field. Our chief aim is to provide a comprehensive starting point to widen perspectives and give practical advice for those who are new(er) to this topic, thus lowering barriers to introduce NGS specifically in HPV-based research and clinical applications.

We are active in the field of HPV testing at the International HPV Reference Laboratory and the Scottish HPV Reference Laboratory (SHPVRL). Both entities were established in 2008, under the auspices of WHO (LabNet) and through the National Health Service of Scotland respectively. As such, we are committed to the evaluation and application of new technologies, including NGS technology, specifically to support the prevention and management of human cancers associated with HPV. In the present work we discuss the key stages of HPV-specific applications utilizing NGS and offer practical suggestions for quality-assurance procedures to support these stages, focusing on HPV whole-genome sequencing approaches. We believe that our experience may facilitate the implementation of NGS in laboratory settings so that it can play an increasing role in research, epidemiology, and importantly clinical testing. With respect to the latter, this is an important consideration, given the increased incorporation of NGS systems into routine departments of laboratory medicine, partly because of national strategies designed to harness the benefits of genomic medicine at the patient level in an agile but comprehensive way [21]. NGS is also consistent with the concept and aspirations of precision medicine, defined as an “approach for disease treatment and prevention that takes into account variability in genes, environment and lifestyle for each patient” [22].

## 2. Materials and Methods

### 2.1. NGS-Process Step 1: Laboratory Procedures

#### 2.1.1. Pre-Analytical Sample Processing

All specimens used for the identification of HPV by existing molecular tests can in theory be used for NGS. However, various biospecimen types may present their own unique challenges. Liquid-based cytology samples, swab samples, or fresh frozen biopsies may typically be readily applied on an NGS platform. However, cells and tissue derived from formalin-fixed paraffin embedded (FFPE) material are more likely to be associated with fragmented nucleic acid and crosslinks between intracellular macromolecules such as proteins and DNA. Fragmentation can be a rate-limiting factor in approaches that demand longer amplicons, which is why it is useful to note that researchers have successfully used shorter amplicons when working with FFPE DNA using NGS technologies [23,24,25,26].

#### 2.1.2. Nucleic Acid Extraction Method

Ideally, extraction methods should be assessed with a pilot panel of samples before embarking on a large project, in order to determine the quality and suitability of the specific extract for downstream NGS. Where possible, we recommend investigators evaluate at least two different extraction technologies to maximize nucleic acid yield. The quality, quantity, and fragment length prior to the library preparation must be determined, as different library preparations may require different nucleic acid input, quality, and length recommendations for library success. For Illumina (San Diego, CA, USA) DNA libraries for example, most of the protocols are optimized for 1 ng of input. Assessing the DNA purity is needed to ensure that the extract does not contain possible contaminants (EDTA, phenol, and ethanol) which can result in assay failure. UV absorbance is a common method used for assessing the purity of a DNA sample, and protocols generally define the “pure”/acceptable range as having an absorbance ratio values of 1.8–2.0 [27,28]. Highly fragmented nucleic acid extraction can lead to missing regions and sections with a low number of reads (low coverage), and the analysis may fail if subjected to enrichment-based amplification methods (see further below) [29,30]. Fragment length can be analyzed using a qPCR with specific length amplicons (same as target amplicon size if PCR-based enrichment is used) or through gel electrophoresis. As described above, a sample being highly fragmented prior to library preparation does not definitively preclude it from sequencing; however, knowing fragment length informs downstream options (e.g., if material is highly fragmented, operators can opt to skip fragmentation steps within the library preparation). HPV positive (with known HPV sequence) and negative internal control samples are necessary to demonstrate that nucleic material has been correctly extracted during the extraction process.

#### 2.1.3. Specimen Enrichment Approach

While HPV-positive clinical samples contain HPV nucleic acid, they are naturally dominated by nucleic acid from non-HPV sources (i.e., human and other-microbiome). If NGS is performed directly on the extract, without a targeted approach, the HPV content is at a relatively low proportion vs. the total nucleic acid sample. As the human genome length is 3 billion base pairs vs. ~8000 base pairs for HPV, the relative proportion of human nucleic acids extracted is +200,000%.

Sequencing studies reveal that viruses typically represent less than 1% of the total genomic material detected in a human specimen [7], and therefore, detection of any virus by NGS formerly required subjecting specimens to either (a) host genome depletion or (b) viral enrichment, first. Different approaches to increase the viral component, include low-speed or high-speed gradient centrifugation, separation of long chromosomal DNA, digestion of nucleic acids not protected by virions (e.g., nuclease treatment), filtration to remove bacterial and host cells, or targeted sequence capture [31,32,33,34,35,36]. Each of these procedures may bias against detection of some viruses; therefore, pilot studies to validate accuracy and reproducibility of the method for the investigator’s specific purpose are necessary. A variety of methods have been described to enhance the HPV content of a sample which are described below.

(A). Depletion Protocols Using Saponin- or Lysis-Based Methods

Saponin is a non-ionic surfactant that depletes the human genome affecting the pathogen-human DNA ratio [37,38]. MolYsis (Molzym, Germany) is a commercial product which works through selective lysis of host cells and associated degradation of released host DNA. Both products thus reduce the amount of host nucleic acid, enrichening the HPV DNA (or other desired bacterial, fungal, or viral DNA) while simultaneously removing potential PCR inhibitors. However, replicating/intracellular viruses are also depleted with these methods, and potential loss of viral signaling can occur, especially for viruses (such as HPV) that integrate in the human genome.

A particular NGS application related to HPV is the detection of viral integration sites into the human genome. When performing WGS, authors have reported that tumors had either a small or a very large deletion in the viral genome and discovered that these deletions were the result of either HPV integration into the human genome or HPV–HPV sequence junctions [39]. It has further been reported that at least 83% [40,41] of cervical cancers with HPV infection have HPV integration, which can occur at any chromosome but more frequently at certain fragile sites [42]. HPV integration can significantly increase related gene expression and has been associated with a worse survival rate (compared to those with episomal HPV) [43,44,45,46]. HPV integration status may therefore have promise as a biomarker for risk stratification [47,48,49], including the monitoring of treatment and therapy. Different methods have been used to study HPV integration sites (e.g., amplification of HPV oncogene transcripts and detection of integrated HPV sequences by ligation-mediated PCR). With the development of NGS, whole-genome sequencing has been used for virus integration sites detection [50,51]. However, it requires large amounts of sequencing data, and thus is not applicable in clinical usage, which requires fast and accurate results. To date, the development of new NGS methods for HPV integration detection with high accuracy and prompt reporting capacity is ongoing [52]. However, the best way currently to detect integration sites is reached by using probe-captured sequencing methods (see section “Enrichment protocols” below). After enrichment of virus genomic material, the fusion fragment of human and HPV sequence is isolated and further sequenced by NGS.

(B). Enrichment Protocols

In addition to depletion protocols, the two most common enrichment protocols for HPV are PCR enrichment in the absence of chemical components and using capture protocols. PCR enrichment involves performing a PCR reaction prior to library preparation. For WGS, this technique may include amplification of the whole genome of the target virus via overlapping primers covering the entire genome (other techniques may not require amplification of the whole genome). A disadvantage of this technique is that there is a need to know the target that is to be sequenced a priori—thus, the method is not valid for detection of novel or nontargeted HPVs. Additionally, in the case of fragmented material, the number of primers required can be high, which requires a structured a priori design approach, and the primers may vary in their affinity for the separate regions. Primer-dimer formation can also limit efficient target amplification, and therefore, pilot studies are needed to validate the protocol for each specific purpose [9,53].

To overcome these bottlenecks, unbiased amplification (not based on PCR) has been commonly used for viral enrichment, as it amplifies all DNA material present in the sample. Multiple displacement amplification (MDA) is the gold standard method for non-PCR-based amplification techniques, where the reaction is based on annealing random hexamer primers to the DNA template [54]. MDA provides an effective way of amplifying minimal quantities of DNA, but there exist biases associated with this technology. Chimera formation, preferential amplification of circular single stranded DNA, and nonuniform amplification of linear genomes have been documented [55,56]. Authors have quantified the amount of amplification of both human DNA and HPV DNA by adding 20 copies/µL of HPV 16 plasmid to samples of human placental DNA at 1 ng/µL and reported an amplification of 26-fold for human DNA and 679-fold for HPV 16 DNA, suggesting that MDA is a good method for enriching circular HPV genomes [5]. Using MDA and NGS sequencing, researchers have been able to detect a plethora of novel HPVs as well as known HPV types, not detected by traditional PCR-based enrichment methods, among skin lesions/tumors and condyloma accuminata [3,4,7].

Another enrichment approach is to use a set of specific probes, “baits”, to recover HPV sequences from the entire genome of the virus. In brief, labelled biotinylated HPV specific probes are captured by streptavidin coated magnetic beads after hybridization, resulting in “pure” HPV-derived reads. Consistent with the overlapping PCR approach, these probes need to be designed in advance but can be modified as required if low read numbers are obtained for some regions of the viral genome. Although it can be expensive, the advantage of the probes/baits approach is the large number of different probes one may include, allowing thousands of probes in one design. This means that it is possible to load the analysis with probes for all known HPVs, depending on purpose. Furthermore, it is the current gold standard method for integration studies [52].

Regardless of protocol applied, to validate the quality of host depletion or viral enrichment, positive and negative control material must be added at the nucleic acid extraction step and carried through the enrichment/capture/depletion step and all stages of subsequent analysis. As a positive control, cell line material infected with HPV is commonly used. However, we would recommend using specimens that contain both human DNA and HPV DNA in the typical concentrations that would correspond to real-life clinical specimens (99:1), as the sensitivity of the different approaches may vary. As a negative control, human DNA (HPV free, but containing the corresponding background “noise” as a true HPV-negative sample) or DNA-free water can be used. Note that if primer-based target enrichment is used, negative controls may contain noise such as primer-dimer bands when assessed by electrophoreses as well as unspecific amplification; these should be clearly discriminated from target sequences. Again, the introduction of homogeneous internal quality controls (IQCs) in the nucleic acid amplification should help in the identification of such issues. Internal positive control material demonstrates whether the depletion/lysis has removed/reduced the HPV target in large proportion during the sample preparation stage. For the enrichment step, positive control material should reflect if amplification of the target regions/genome occurs and negative controls help determine whether any nonexpected amplification/targeting occurred.

#### 2.1.4. Direct Sequencing

Several operators have opted for performing WGS directly after nucleic acid extraction, without enrichment. As described above, reaching a high sequencing depth is required, due to the low proportion of viral sequences typically present in the human specimens. Direct sequencing has enabled an agnostic approach to DNA presence in clinical samples: while around 10% of cervical cancers are found to be negative for oncogenic HPVs by traditional PCR-based genotyping methods [57,58,59,60,61]; direct sequencing can reveal the presence of viral sequences of potentially causative HPVs in such cases [13,62]. Interestingly, most of the HPVs detected with direct sequencing among the carcinomas negative by traditional PCR typing systems corresponded to HPV types within the explicit detection range of these assays. Therefore, the information obtained by direct sequencing can be used, not only to detect novel variants or types that may have escaped traditional amplification but also as a way to quantify and monitor shortfalls in detection due to sensitivity issues. Furthermore, evidence suggests that HPV-negative cancer patients have a worse longitudinal prognosis [60,63,64]; this is reflected in the staging system for oropharyngeal cancer which acknowledges the dichotomous disease status based on HPV presence [65] as does the recent WHO update for female genital tumors [66]. How “best” to annotate HPV status in cancer tissue is an area which arguably lacks consensus in the literature; however, NGS provides a powerful tool to at least resolve which cancer cases may be truly virally negative.

#### 2.1.5. Library Preparation and Sequencing

At present, there are several different sequencing chemistries available. Each system has its own protocols and due to the diversity of platforms, and rapid pace of developments, we cannot recommend a specific one for all HPV applications. Akin to the assessment and introduction of any new technology in-house, we strongly recommend validation that includes confirmation of expected results from “known” quality materials and the evaluation of different kits with the specific analytical purpose in mind. For laboratories looking to embed NGS into the accredited scope of their clinical service, initial validation followed by yearly verification would likely be mandatory.

Quality, quantity, and fragment length analysis is a must to confirm success of library preparation. Library preparation protocols usually inform the operator about the concentration and size expected for prepared libraries. If measurements do not reach the expected values, (e.g., fragments are too big/small or the library concentration is too diluted) optimization of fragmentation times, clean-up processes, or amplification steps should be performed. Larger fragments cluster less efficiently than smaller molecules, and a low concentration of prepared libraries translates into a low number of sequenced reads. Here, it is crucial to consider in which context libraries are analyzed; in a research study, suboptimal measurements can occasionally be acceptable but probably never in a clinical context where protocol adherence is paramount and actionable test results are needed.

One also needs to perform accurate normalization to obtain homogeneous distribution (number of sequencing reads) of the samples and assure that the proper sequencing read length is used, depending on the insert size of the library. As an example, Illumina libraries prepared with dual-indexing that show a fragment length of 200 bp should not be sequenced with 2 × 150 bp, as part of the fragment length (around 130 bp) corresponds to adapter sequences, and the insert size, which is the actual query sequence, only comprises 70 bp (200 − 130 bp). Thus, 80 bp of the 150 bp sequenced (150 − 70 bp) does not contribute useful information. Libraries from positive and negative samples must be added into the final input dilution. Our recommendations for quality-control steps in the NGS workflow are summarized in Table 1.

### 2.2. NGS-Process Step 2: Bioinformatical Analysis

#### 2.2.1. Raw Sequence Data Management

The output data from a sequencing machine are often referred to as raw data. Raw data management generally includes filtering steps to remove poor-quality data and host-derived human reads and continues by mapping nonhuman high-quality reads directly to a known reference database or performing a de novo assembly approach, finishing with HPV taxonomy classification, phylogenetic analysis, and variant calling. There are several open-access tools that can be used to analyze “big data”. A set of bioinformatic algorithms, when executed in a predefined sequence, is collectively referred to as a bioinformatics “pipeline”. These pipelines can be designed in-house by teams with available bioinformatics expertise or obtained as ready-to-use applications from commercial suppliers. The premade pipelines are principally aimed to users with little bioinformatic experience and act as a “blackbox” (user does not know which algorithm and calculation(s) are used by the pipeline).

#### 2.2.2. Sequence Analyses: Quality Assessment of Reads

Each bioinformatical tool deployed in the pipeline uses different algorithms and parameters when handling data. When the objective of a project is to resolve a 0.5–1% difference between sequences, these differences in parameter settings could mean a different interpretation on nucleotide/mutation level is reached depending on which pipeline is being used. Ergo, two studies on the same nucleotide position could reach two different conclusions as to whether a mutation is present.

Errors due to poor sample handling and storage conditions, polymerase bias, PCR- or qPCR-induced errors, and incorporation errors within sequencing may be introduced during sample preparation, amplification, library preparation, and sequencing stages. While these errors might not interfere with the identification of an HPV type (where the sequence divergence is 10% relative to its most closely related type), they might compromise the identification of sublineages or variations in nucleotides that could have implications on accuracy, consistency, and, potentially, predicted phenotype. Careful sample handling, selection of a high-fidelity polymerase [67], and quality control of the raw data is a must [68].

Most analysis applications use FASTQ files as input for analysis; however, different sequencing instruments may give different extensions for raw sequencing data (e.g., Illumina generates bcl files), and the first step is the conversion of those files to a standard format (FASTQ). Platforms usually provide software for the desired conversion (e.g., bcl2fastq from Illumina). Raw FASTQ files should be subjected to quality trimming and adaptor removal as a first step. Software for quality trimming and adaptor removal include Cutadapt, Trimmomatic, Trim Galore!, SeqTrim, and FastX among others [69]. Quality trimming is performed to remove low quality reads and aims to reduce the effect of the progressive decrease in sequencing quality with the increased length of the sequenced library. Trimming removes low quality portions of NGS reads while preserving the high-quality part of such a read. The user can specify the quality cut-off for a base or use a “sliding window” approach (defined as setting a cut-off for the average quality detected in a number of X contiguous bases instead of just one base). Quality is usually checked according to the Phred quality scores, which are scores logarithmically related to base-calling error probabilities [70]. As an example, a Phred quality score of Q30 corresponds to a base calling accuracy of 99.9% (1 error per 1000 bp). The minimum quality recommended in the literature is a Phred quality score of 20 (99.0% accuracy; 1 error per 100 bp), with the optimal quality however being above Q30 [71].

#### 2.2.3. Alignment of Reads to a Suitable Human Reference Genome

To obtain a dataset that contains only reads of interest, e.g., viral-related reads for HPV detection, nontarget sequences may be filtered out at the bioinformatics level to speed up downstream analysis and decrease the risk of misassemblies of genomic data. Most researchers applying WGS from sequenced extracted material opt for filtering out human genome sequences only (leaving HPV plus “other” microorganism reads). This is practical as human reads (according to our experience of multiple sequencing projects with a metagenomic perspective) account for approximately 90% of the total (with some variability depending on the sample origin) [7,13,62]. While several successive “versions” corresponding to human genome reference exist, it is recommended that the latest build (released in December of 2013), officially named GRCh38 (Genome Research Consortium human build 38) or commonly Hg38 (human genome build 38) is used.

Numerous aligners exist so far and are being developed in order to achieve greater accuracy pertaining to precision. Widely used tools include BWA-MEM (Burrows-Wheeler aligner) [72], SOAP2 [73], or Bowtie 2 [74], but several commercial tools are also available such as NextGenmap and Novoalign [75,76].

While some operators may, at this point, choose to filter out reads that are identical to the human reference genome (100% identical), another approach is to employ looser parameters which accept reads as human if the identity and coverage across the human genome sequence of interest are at least 95% and 75%, respectively. This latter approach allows for the detection of possible mutations not anticipated in the reference sequence, although this flexibility should be tempered so as not to misclassify nonhuman reads as human. An important thing to consider after performing the aligning/mapping to the human reference genome is to select which “unmapped” reads are to be used in downstream analysis. Sequencing with paired-end reads may contain (1) paired-end reads where both reads are unmapped, (2) paired-end reads where one of the pair read maps to the genome and the other does not. Operators may decide to discard nonhuman single reads and continue only with nonhuman paired-end reads, or to include them all; such a choice is entirely dependent on coverage obtained and the aim of the project/resolution required.

#### 2.2.4. Alignment of Reads to a Suitable HPV Reference Database

Once human (or host) sequences are filtered out from the high-quality data set, most operators align reads to a known and curated HPV database for HPV classification or to the reference HPV genome in question. Currently, there are 222 different HPV types officially established (data accessed on 18 March 2021 from Hpvcenter.se) and another 220 putative novel HPV types (not cloned and investigated by the International HPV Reference Center) whose complete sequence can be found in the public database from the papillomavirus Episteme (data accessed on 18 March 2021 at https://pave.niaid.nih.gov/).

Furthermore, there are many partial genomic sequences of HPV isolates (not all specifying which HPV type they correspond to) available at public databases, with GenBank having >33,500 hits retrieved when typing “human papillomavirus” (data accessed on 18 March 2021). A recent publication [77] detected up to 0.5% chimeric sequences and/or taxonomy errors when analyzing HPV sequences in the GenBank database. This highlights the importance that the database be obtained from a quality reference repository and that local curation is performed before doing any type of alignment, such as checking for potential errors and updates. In-house databases belonging to individual investigators should be periodically updated with canonical or reference types, as new HPV types are continuously being discovered [5,78].

It is particularly key to note that, when aligning the sequencing reads to a specific HPV genotype, operators ascertain that the correct reference sequence is used. There are 3650 sequences belonging to HPV16 isolates in GenBank (sequence length 7500–8500, data accessed on 18 March 2021), showing differences that may reach up to 10% of the total genome. If each separate investigation were to use a different sequence as reference genome, then comparison between publications becomes challenging at best. The reference genomes that should be used for each HPV type are provided at the International HPV Reference Laboratory website (Hpvcenter.se); accessed 7 July 2021, as well as at the papillomavirus Episteme database (https://pave.niaid.nih.gov/; accessed 7 July 2021). The latter resource has a contemporary collection of internationally ratified sequences from the reference clones corrected for known sequencing mistakes in the original sequences.

#### 2.2.5. Identification of HPV Types/Lineage/Sublineages

Classification of HPVs is based on the nucleotide sequence homology of the *L1* gene, which is the most conserved region of the viral genome. Within the family, different genera share less than 60% nucleotide similarity. Within each genus, different species share between 60% and 70% similarity. Below the species level, a novel HPV type shares less than 90% similarity to any other type [79,80,81]. The definition of a variant lineage is that the L1 open-reading frame differs by more than 1%, but less than the 10% that would make it another HPV type [82]. A variant sublineage is defined as groups of sequences with 0.5–1.0% differences between genomes [83].

There exist different tools for the identification of variants. One of the most used and user friendly is BLAST [84]. This tool compares the sequence under investiagation to sequences stored in the database, detailing statistical significance of matches. Again, the importance of using standard references for HPV variants is essential. Burk et al. described the representative genomes for viral variant lineages and sublineages, and most authors rely on these sequences as variant lineages references [82].

If a phylogenetic analysis is required, different open-source tools exist (RaxML, MegaX) that infer phylogenetic trees after choosing the statistical method [85,86].

#### 2.2.6. Evaluation of Coverage across the Genome

If the purpose is to detect HPV genotypes (not within-genotype specific variant calling), once the reads are aligned to the HPV database, we recommended that cut-offs are applied on which HPV positivity is based (Table 1).

This could be, e.g., setting a minimum of 10 reads detected for a specific HPV type together with a coverage of at least 10% of the HPV genome (around 800 bp coverage). This approach would avoid false positivity generated by background noise (e.g., presence of many low complexity reads mapping to just a small region of the genome). If phylogenetic analysis or variant calling is required, a FASTA file with the “query” sequence must be created. When creating a FASTA file from the obtained sequencing reads/contigs, investigators should be aware of the extent of genome coverage to see if there are missing regions

Evaluating the full coverage of the sequence is important, as several tools that convert the sequencing reads into a FASTA file use a reference sequence to account for the regions that are not covered. The use of “N”s is recommended for the positions that are not covered by the sequencing reads. For variant lineage assignment, exclusion must be considered for specimens with poor read depth (<200 median depth) and/or low genome coverage (<80% genome coverage) [53]. For variant calling, even stricter cutoffs should be applied. Additionally, further steps including marking duplicates to identify read pairs likely to have originated from duplicates of the same original DNA fragments and recalibration of base quality scores should be performed, as suggested by the best practices at GATK [87]. Considering just the base depth as a cutoff for variant calling (e.g., five reads per position) is not enough to assure accurate calling. It is essential to differentiate between true positive variants and false positive variants. Parameters and statistics which describe how many reads cover the variant, what proportion of reads are in forward vs. reverse orientation, and what the sequence context is like around the variant site should be considered.

#### 2.2.7. De Novo Assembly of HPV Contigs

When the correct reference genome is not known, the (re-)construction of the sequenced genome must be performed without a priori knowledge of either the correct original sequence (or the order of the DNA fragments), by assembling overlapping reads into one or more contigs. This process is known as de novo assembly. Subsequent post assembly assessment is mandatory to reduce the risk of chimeric sequences and possible miscalling of HPV positivity in samples and/or erroneous calling of new HPV variants/genotypes. HPV-Chimera scripts exist to help researchers determine the accuracy of their HPV contigs [12,77].

#### 2.2.8. Digital Quality Assessment

While positive and negative controls can be incorporated into laboratory experiments and several quality check-points are available during the whole laboratory process, we currently lack an agreed approach for the quality control of bioinformatical tools and pipelines. Digital IQCS can be prepared from confirmed and verified positive material and stored as FASTQ files (raw data). Interlaboratory exchange of data can provide reassurance by comparing results on sequences derived from the same specimens. However, this requires resources and collaboration which may not always be available in the short term. Therefore, it would be beneficial to have positive IQCS available in an online repository that could be used to verify the pipeline when setting up a new service/test or when a tool is updated.

#### 2.2.9. Journal Submission Requirements

Recently, several journals have started to request that all authors who submit manuscripts containing NGS data provide a detailed summary of sequencing coverage and quality statistics. For example, the International Journal of Cancer requires a summary from submitting authors that must include all information about library preparation, sequencing technology information (e.g., platform, read length, and paired-end/single read approach), as well as preprocessing, quality control, and filtering of the raw NGS data [88]. Furthermore, the sequencing coverage and quality statistics of each sample must be summarized as a Supplementary Table.

#### 2.2.10. HPV NGS in Clinical Settings

Application of NGS for clinical testing requires a level of quality assurance and monitoring likely to be even more stringent than systems set up in research laboratories. Verification and validation for each of the steps that make up the NGS process is the key to obtain and provide reliable results to clinicians and patients. Any minor change of the wet-lab protocol or any parameter in the data analysis requires a full verification with previously known samples/sequences. This in combination with the external quality assessment, and accreditation helps ensure the validity of the clinical results.

#### 2.2.11. External Quality Assessment and Accreditation

Suppliers of EQA schemes have developed external quality materials to support NGS sequencing results for various pathogens and human genes (e.g., GenQA, Statens Serum Institut, and QCMD (pilot)). At time of publication, we are not aware of any official HPV EQA scheme to support NGS for wet and in silico analysis or indeed data/dry analysis. This is arguably a current deficit, as while interlaboratory exchange of materials is undoubtedly helpful for quality assurance and validation of HPV NGS, such exchanges do not wholly “stand in” for consistent performance in a formal accredited EQA scheme(s). Should HPV NGS move to the diagnostic context, then this would increasingly require address.

#### 2.2.12. Data Storage Requirements

Data storage demands of NGS are often very large and need to be carefully considered before local implementation. Unfortunately, there is no (international) consensus on what and how data should be stored beyond the general recommendation that it should be stored in line with national and local capacity and governance policies. In high-level terms, we recommend storing the raw FASTQ files in a compressed mode (.fastq.gz), the final output, and the full-log file (documentation on the software/tools used, including versions, parameters, and github location, needed to obtain the output files) making the whole analysis reproducible.

If intermediate files are to be kept, they should be stored as standard open-file formats FASTQ, BAM, and VCF, facilitating the exchange with other laboratories, where governance permits. Cloud-based storage could be a very helpful tool; however, this may be challenging to reconcile with data protection. Currently, most journals ask for data availability and request that authors upload all nonhuman sequences detected in the study to different databases (such as the European Nucleotide Archive (ENA), Sequencing Read Archive (SRA), and Genbank) to make research publicly available and re-usable for other scientists without compromising confidentiality.

## 3. Discussion

Next-generation sequencing (NGS) has enabled researchers to detect human papillomavirus (HPV) infections with unprecedented sensitivity and accuracy while simultaneously providing the whole viral sequence to be analyzed for viral point mutations, variant lineages, and genome variations. Increasing incorporation of NGS into laboratories for research, epidemiology, and diagnostic purposes may be only a matter of time, particularly as costs reduce with increasing demand and competition.

Certainly, the use of NGS for clinical workstreams generally requires accreditation/auditing from an independent regulator. As an example, the National Accreditation Body for the United Kingdom (UKAS) now has expertise to assess and accredit labs that have NGS in scope, working to ISO 15189:2012 standards. This process covers assessment of staff training, quality control of pipelines, initial validation of the whole process compared to a gold-standard approach, and reproducibility of results plus checks to ensure security of data and access is correct. The magnitude of the effort to achieve accreditation in a particular service laboratory is likely to depend on local support for the integration of NGS to support public health epidemiology and precision medicine in general terms. The SARS-CoV-2 pandemic has brought about an exponential increase in molecular testing and associated infrastructure, including that required to support sequence-level variant detection. This is likely to pay dividends for the establishment of “cross organism working” to a diagnostic standard [89]. Certainly, NGS has the potential to support risk stratification of patients with HPV-associated disease through its ability to detect types within tissue with exquisite sensitivity and through providing subtle insights into aspects of HPV infection that may be predictive of outcome (integration pattern and status, delineation of dominant variant(s), and viral load). NGS may also be applied to liquid biopsies, from blood, to support longitudinal monitoring of treatment success [90].

The bulk of HPV sequencing to date has been performed using the IonTorrent initially [9,11,91], followed by the newer Illumina platform [7,53,57]; some very recent studies have also used nanopore platforms [52,92]. While many studies report higher sensitivity and accuracy when comparing NGS to routine genotyping methods [6,8], there are very few studies where one HPV NGS approach has been directly compared to another in a head-to-head approach using the same sample set [93,94]. Hence, there are fewer data available compared to the relative wealth of peer-reviewed literature on head-to-head performance of traditional HPV molecular assays. While many researchers are already using NGS for detection and analysis of HPV-associated diseases, there is no clear international consensus about what steps are to be performed nor which quality criteria are appropriate.

Typically, published presequencing laboratory protocols appear well validated for providing detail on clear-quality assessment procedures and specifying the requirements needed for library preparation evaluation and success. Nevertheless, even though the inclusion of positive and negative controls should be a must, the presence and/or description of these controls is not commonly found within NGS publications. This may be in part due to the relatively high cost of NGS. Hornung et al. reviewed ~265 publications which had used NGS for microbiome research and found that only 30% of publications used any type of negative controls and that less than 10% reported positive controls. Additionally, they observed that some of the results reported were potentially indistinguishable from contaminants [95].

Furthermore, to the best of our knowledge, quality assessment of bioinformatic analysis appears not to be as standardized, when compared to the analytical sample handling stages. However, we consider it key that the use of a curated and updated databases, use of standard reference genome sequences, and description of the parameters used for each step should be described fully in all publications.

The present piece aims to describe and summarize the application of WGS for HPV detection and has mainly focused on DNA detection to detect the whole HPV sequence. Nevertheless, RNA sequencing should not be forgotten as an alternative method for HPV detection, as RNA transcription not only enables detection of HPVs but provides further information on the active HPV infection that drives viral oncogene expression. In the cancers that are known to be caused by HPV, transcription of viral genes is necessary for viral pathogenicity [96]. Studying transcription of the E6 and E7 oncogenes has been useful to elucidate which infections are likely to be involved in the etiology of the tumor, e.g., in head and neck cancer studies [97,98,99]. Even though RNA sequencing is not usually performed within studies aiming to analyze the whole HPV genome (as noncoding regions, e.g., URR, are not detected if RNA extraction and sequencing are performed), important information about active infection and viral oncogene expression is obtained with this sapproach.

International collaboration is essential to efficiently further knowledge, scientific development, and concerted efforts to combat globally prevalent viral infections. In the case of HPV, the International HPV Laboratory Network LabNet was created by WHO in 2006 to support global development of laboratory standardization and quality assurance of HPV detection methods. LabNet concentrated on evaluating and improving methods used for research and evaluation of HPV-based screening and vaccination [100]. As part of this effort, LabNet published an HPV laboratory manual, based on knowledge and experience gained through international collaborative studies, aiming to assist in establishing the laboratory support required for HPV research [101]. The successor to LabNet, the International HPV Reference Center (Hpvcenter.se), aims to support reliable and comparable HPV detection services, allowing data to be internationally comparable. The Center has organized and issued global proficiency panels (sets of blinded samples containing HPV genotypes at different concentrations) since 2008, and a definite improvement in average assay performance globally has been seen since the panel was issued [102]. The SHPVRL has also acted as a hub laboratory to support the creation of materials and best practice documents to facilitate the introduction of new HPV technologies and their continued monitoring [103,104].

Together. we now work between our two laboratories to exchange samples, know-how, and protocols for bioinformatical flow and sequence analyses. Hopefully, this will strengthen the quality of work produced by both settings and act as a catalyst/model for future international endeavors. We propose that it is time for NGS to be included in the global efforts on quality assurance and improvement in HPV-based testing and diagnostics. By establishing a set of quality standards and best-practice statements, the community could systematically develop and apply NGS guidelines suited for HPV research, epidemiology, and diagnostics to ensure this innovative and powerful technology is developed in an internationally comparable and robust manner.

## Figures and Tables

**Table 1:** Steps, potential quality issues, and proposed mitigations for next-generation sequencing (NGS) analytical workflow, with a focus on whole-genome sequencing purposes.

Human Papillomavirus Detection by Next-Generation Sequencing		
NGS Step	Possible Difficulties	Mitigations
Sample preparation	Nucleic acid quality and/or quantity outside library prep kit requirements	Selection of appropriate nucleic acid extraction methods. Pilot study comparing different extraction kits. Selection of enrichment or depletion protocol. Introduction of homogeneous internal quality controls Electrophoresis, bioanalyzer, and/or fluorometric quantitation. Selection of further protocols based on the fragment size (e.g., use of shorter amplicons if DNA is highly fragmented)
	Incorrect fragment size (too short or too long)	
Library preparation and sequencing	Incorrect fragment size (too short or too long) Incorrect number of sequencing reads or partial reads	Correct selection of library kit and fragment length. Correct selection of sequencing kit (e.g., 75 bp and 150 bp) to avoid sequencing adapters or longer fragments that insert size.
Data analysis	Low sequencing depth	Library preparation and sequencing piloting, and re-analysis Confirm reference sequence is correct. Use of updated database. In case of low sequencing depth at the beginning or end of the reference sequence, note that HPV is circular and not linear as the reference. Confirmation that desired alignment cut-offs are correct.
	Incorrect alignment	
	Mix/chimeras of microbial organisms	Filter reads—use of updated databases and careful settings of parameters. De novo assembly evaluation (HPV Chimera scripts)
	Validation of pipeline	Digital IQC, EQA, external assessment. Interlaboratory comparison
Storage	Large amount of data	Cloud services, compression of files, and storage of only raw input and final output.
	Security	Restricted super-user access, individually curated data access Analyst working only with coded/pseudonymized samples (where an independent database administrator holds the key code at another site)
	Length of data storage	Organization policy/data archiving laws and regulations

## Data Availability

Not applicable.
